# Quantitative evaluation of family functions and changes in a postmodern context

**DOI:** 10.1016/j.heliyon.2021.e07435

**Published:** 2021-06-29

**Authors:** Catya Torres, Diego Vallejo-Huanga, Ximena Ramírez Ocaña

**Affiliations:** a Universidad Politécnica Salesiana, Department of Psychology, GIFE Research Group, Quito, Ecuador; b Universidad Politécnica Salesiana, IDEIAGEOCA Research Group, Quito, Ecuador; c Universidad de las Américas, Department of Physics and Mathematics, Quito, Ecuador; d Universidad San Francisco de Quito, Department of Mathematics, Quito, Ecuador; e Universidad Politécnica Salesiana, Department of Psychology, GIPS Research Group, Quito, Ecuador

**Keywords:** General systems theory, Family behavior modeling, APGAR, Social system, Dysfunctional family

## Abstract

Society lives the transition between modernity and postmodernity. In this context, the family is considered as a fully dynamic system that changes over time. Therefore, family structures are in constant motion, and family functions also require changes. Sometimes the functions in the family change, but the structures do not, so conflicts within the family may appear. The objective of this research is to show how families evaluate their role in the postmodern context. Hence, the evaluation was carried out in 37 families through the APGAR test, a questionnaire that explores five areas of family function. Statistical analysis and data processing were performed with free software tools and the experiments may be reproduced as the data and code are hosted in open repositories. The results show that the perception of dysfunction at the individual level does not differ from the family perception. The families typically deny the conflict and the implicit changes in the family's functions because they do not know how to handle these changes. Likewise, the study shows that the changes in the family, attributed by several authors to the transition related to the postmodern paradigm, outline a growing trend towards the perception of the dysfunctionality of the family system over time.

## 1 Introduction

The postmodern context implies changes in different levels of organization in society, especially in institutions. The family, as an institution, is one of the most affected by the transition between modernism and postmodernism. The postmodern context has implied changes in western societies and has promoted the destruction of central arguments in language, identity, culture, by using mechanisms of dispersion and uncertainty [1]. This paradigm is understood to include new societies in constant transformation and for its maintenance, it is intended to increase the flexibility of social structures. Thereby, the fragility of institutional life and calling into question the stability discourse is exposed [2].

The modeling of social systems has been widely explored from the point of view of social systems theory [3], [4], [5], [6]. Although there are several approaches to conceptualize the family [7], experts agree that there is no fixed system due to its complexity, interactions, and structure. Despite this, several studies have tried to model the behaviors and iterations that occur inside [8].

In quantitative processes, a system refers to a set of structures or parts that interact by means of a set of relationships [9]. From the perspective of the general theory of systems [10], the family is considered as a dynamic system that is subject to a continuous establishment of rules and search according to them [11]. In recent years, this perspective has been used to quantitatively understand the structural functioning of the family and their inter-relationships [12].

Within family, changes are frequently caused by the way of thinking of its members and its different lifestyles. The traditional structure of the family is questioned by the speeches, which emphasize cultural diversity. An uncertain world, where people must develop capacities to handle chaos, demands flexible and dynamic structures, which can be modeled through systems. The family, as a relational system that interweaves its discourse from the experiences of each of the members, is affected as an institution by the transition between modernism and postmodernism. Such effects are evidenced more clearly in the conception of marriage, socio-labor roles, and education.

The 1950s were known as the golden age for the traditional family because it referred to a model that anchored a married heterosexual couple with children, with low divorce rates and a strong specialization of family roles [13]. The postmodernism and its effects, such as unemployment, longer life expectancy, and smaller family size, have blurred the roles of its members.

The conception of marriage was the boundary between adolescence and adult life. In contemporary times men and women leave the nest, supported financially by themselves, living with a partner, and sometimes having children without marriage. This behavior seems to diminish the importance of a wedding as a ritual that indicates the beginning of adulthood [14]. Likewise, the fact of getting married today denotes a different conception, a commitment of loyalty and fidelity that, the human being is not willing to assume because it is marked by a liquid modernity orchestrated by consumption [15]. The increase in divorce rates appears as an increasingly evident phenomenon in the configuration of postmodern family structures in the Latin American context. Pavan [16], in an analysis of the postmodern family in Argentina, points out that from the 1960s on, the attribution of authority within it became complex, in correspondence with the increase in divorces, separations, and conjugal re-composition. In Ecuador, the first registry that is included statistics of marriages and divorces corresponds to 1965 and in this registry, there are 30362 marriages and 1300 divorces. Between 2006 and 2016, divorces increased by 83.45%, while marriages fell 22.01% [17].

Gershuni et al. [18], in their study on the evolution of the uses of time, between 1960 and 1985, in six industrialized countries (United Kingdom, United States, Canada, Denmark, Holland and Norway), found an increased time spent by men on domestic work. Men have become involved in household responsibilities, taking on responsibilities that did not correspond to them socially in modern times, such as caring for minors or older adults, although yet female work prevails [19]. In Ecuador, childcare falls heavily on women, the study of the National Institute of Statistics and Censuses (INEC) on unpaid work in the home states that only in 15.4% of cases is it done by men. Today, motherhood is less and less important in a woman's adult life as it becomes recursive to look at families with only one child or couples without children [20].

The postmodern family is based on the association which is the result of the postponement of paternity, economic independence, with individualized characteristics where pets are part of the family structure [21]. Also, postmodernism contemplates new family structures. The postmodern family encompasses many different agreements, two-parent working families, single-parent families, adoptive families, newly married families, as well as families of gay and lesbian parents [22]. The GLBT family understands that the process of family empowerment, where social norms with respect to gender and parenting influence are deconstructed and then rebuilt [23], consider that the education starts from combining other strategies, as artificial insemination, or adoption between people of the same gender. Families can extract meanings that are anchored in consumption to create a family identity [24].

Regarding the conception of education in postmodernism, there is a philosophical confusion between knowledge, power, and desire [25]. This conception contributes to social changes but does not prioritize cultural values. The family reflects the structure of the social system and as such must be analyzed from different perspectives that promote a better understanding between its different elements and its members' interactions with different institutions [19].

The new family structures, in the post-modern paradigm, imply a redefinition of functions in family contexts, so that family functions of adaptation, partnership, growth, affection and resolve abilities have changed because of the transition between modernism and postmodernism [26]. Several authors have already affirmed that the transition towards the postmodern paradigm increases the perception of the dysfunctionality of the family system [27], [28], [29], [30], [31].

It is important to evaluate family functions to understand how families assimilate changes in postmodern contexts. Studies associate family functions with the management of family relationships, communication skills, and risk behaviors [32]. Other authors propose that family function is related to mental health and could increase the quality of life through the development of resilience [26]. The authors suggest that the inability to understand changes in family functions probably does not allow for the consideration and identification of dysfunctional or symbolic behaviors. Consideration should also be given to how symptoms modify family functions and how changes in functions create new symptoms in family contexts. This happens because the family does not want to accept the changes, so they resist the change and perceive themselves as dysfunctional structures. Finally, studies suggest that interventions are required to improve family functions and help them adapt to the new context [33], [34].

Although there are several works related to the quantitative evaluation of the family and their functions, the changes that these products in the postmodern context, have been little explored in Latin America. In this sense, the Adaptation, Partnership, Growth, Affection and Resolve (APGAR) test allows the family to be understood from its five components. Adaptation: sharing resources and the degree of satisfaction with the care received; Partnership: refers to family communication and joint decision-making on problem-solving; Growth: achieves emotional growth due to the freedom to change roles within the family; Affection: the individual's satisfaction regarding intimate relationships between family members and family interactions; and Resolve: sharing time and satisfaction with the commitments that family members establish [26].

Even though there is empirical evidence from studies carried out with the APGAR test and with families in Colombia, these studies are directly related to families as support in disease processes, the coexistence of families with delinquent adolescents, pregnant adolescent women, or relatives who are going through catastrophic illnesses. It is important to know that there is a scientific information gap regarding postmodernism and its direct effects on family dynamics.

This article quantitatively examines the way in which families evaluate their functions, by means of the application of the APGAR questionnaire [35], in the context of the Ecuadorian family structure. This paper aims to explore the changes produced in families, from the internal perception of their members, without considering the qualitative aspects. Family structure and family functions were considered as variables for exploration since they are parameters around which several authors have indicated changes in the postmodern context [36], [37], [38], [39].

## 2 Materials and methods

### 2.1 Design

The process was designed to examine changes in families and understand changes related to the structure and functions of the family in a postmodern context. In general, the research design used for this research includes a research question that is analyzed and answered using qualitative and quantitative techniques and that also has components of action-participatory research and participant observation.

This research applied an observational study with families in Quito-Ecuador from January 2018 to January 2020 and was conducted with the help of mental health professionals. The study combined qualitative and quantitative data; in this article the quantitative data was processed.

In the Ecuadorian context, people are of legal age when they are 18 years or older. Therefore, for these two segments of sub-populations within the family, two different types of processes were designed, both in the design of the APGAR test and in the way the professionals approached the participants.

Observation, interview, and writing techniques were involved in the research process with families. After designing the process, the professionals who carried out the home visits were trained on data collection, data processing, data analysis and report writing.

### 2.2 Participants

Around 20 families were convened per year, but only 15 families accepted to participate in the process. The sample was made with 15 families per year, i.e., 45 families participated in the entire process. After performing the analysis of the data and due to inconsistencies in its collection, the data of 11 families in 2018, 13 in 2019, and 13 for 2020 were considered. The approximate intervention time for each family that participated in the process, was two months.

Regarding adherence, the process was carried out with 45 families and only 3 families did not finish the process, i.e., 6.7% of the total sample. During the process, demographic information of the individuals, such as: age, gender, familiar role, and location was collected. All the participants voluntarily reported their self-identification of gender, being able to code the variable as a nominal qualitative with only two categories: male and female. The familiar role variable refers to the self-identification of the played by the individual in the family and the following classes were recorded: father, mother, son, daughter, nephew, grandmother, and stepfather. The location variable refers to the geographical site of the family home, with 19 closed categories, that correspond to the territorial organization of the Quito city.

Over three years, this study has been conducted on 77 individuals, with an age range of 7 to 79 years. The mean age of the sample is ${\bar{\mu }}_{s}=38.23$ years, with a standard deviation $\sigma_{s}=16.11$. A prevalence of the gender female 57.14% over the male 42.86% has been identified. Of the total of participants, 71 were adults with age ${\bar{\mu }}_{a}=40.65\pm 14.36$ years, while the remaining 6 were underage people (${\bar{\mu }}_{a}=9.67\pm 2.25$ years). The age range of adults varies from 19 to 79 years and that of underage people between 7 and 12 years. The participants belong to 37 families from different social strata in the Quito city, which were selected through random and non-probabilistic sampling, because the purpose of the study was to obtain deep and intimate information about the families. All participating families were of Ecuadorian nationality.

To select the families, the team of volunteer psychology professionals in charge of collecting the data, carried out an opinion poll in some sectors of the city through institutions or community groups and looked for families who freely and voluntarily wish to take part in the process accompaniment.

The criterion used to choose the participating families was that they were linked to the Universidad Politécnica Salesiana through non-governmental organizations or civil society organizations with which the university has agreements. Families located in the southern, central, and northern sectors of Quito city were searched, and it was trying not to select families from the same neighborhood in order to have a diversity of contexts. Territorially close families were excluded from the sample. In addition, only stable families in the territory were selected, that is, families who were not migrants or who were temporarily in the neighborhood of residence, to avoid leaving the accompanying process unfinished. Those families with members living outside the city or territory were also excluded. Families with members with chronic illnesses, addiction problems, or some type of pathology were not chosen. Nor were families linked to or referred by health systems chosen. It was sought that there is at least one family from each stage of the family life cycle, i.e., families with young children, families with adolescent children, families with young children, families with adult children, and elderly couples. The participation of nuclear families, single-parent families, and extended families was also sought.

### 2.3 Materials

To describe the degree of family dysfunction, the APGAR test was used as a measurement instrument. The APGAR design used a modification of [40] adapted to the Ecuadorian context. This is a standardized and normalized version of the APGAR questionnaire. The validity and reliability of the APGAR family questionnaire on family function have been widely discussed by several authors [41], [42], [43] and it has proven to be a robust and complete tool to assess the way family members perceive the level of family dysfunction globally.

The APGAR test is a tool proposed as an instrument for primary health care teams, in their approach to the analysis of family function, based on the premise that family members perceive the functioning of the family and can manifest the degree of satisfaction with the fulfillment of its basic parameters. Therefore, the APGAR has some limitations since it only examines five aspects within the family; some research questions its sensitivity and practical utility [44], [45], [46]; it can generate bias since it is applied by questionnaire and has a great dependence on the level of education of the participants.

Thus, the APGAR questionnaire contains five questions *Q* (rows *i*) and five possible answers *A* for each question (columns *j*), in a matrix structure. This means that the execution of the APGAR test, per se, were coded in the form of a tuple, question-answer ($Q_{i}-A_{j}$). Only one tuple should be checked in each question, with a response score ranging from 0 to 4 points.

Each $Q_{i}$ of the APGAR questionnaire (Table 1), represents one of the five components of the test (adaptation, partnership, growth, affection and resolve) defined by Smilkstein, respectively. The five possible answers, for each question, were designed with different weights based on a linear symmetric Likert scale, and with the same ratings for adults and underage people. The set of questions ($Q_{i}$) for adults and underage people, and the Likert scale weighted answers ($A_{j}$), offered for the participants are summarized in Table 1.

**Table 1:** Apgar questionnaire: set of questions *Q*_*i*_ for adults and underage people, and their answer options *A*_*j*_ weighted in Likert scale.

Questions for adults (*Q*_*i*_)	Questions for underage people (*Q*_*i*_)	Likert scale weighted answers (*A*_*j*_)
Q1: I am satisfied with the help I receive from my family when I have a problem or need.	Q1: When I am worried about anything, I can ask my family for help.	Never (0 Points)
Q2: I am satisfied with the participation that my family gives me and allows me.	Q2: I like how my family talks and shares their problems with me.	Almost Never (1 Point)
Q3: I am satisfied with how my family accepts and supports my desire to undertake new activities.	Q3: I like how my family allows me to do the new things I want to do.	Sometimes (2 Points)
Q4: I am satisfied with how my family expresses affection and responds to my emotions, such as anger, sadness, love, etc.	Q4: I like what my family does when I am happy, sad, angry, etc.	Amost Always (3 Points)
Q5: I am satisfied with how we share in my family: a) time to be together, b) spaces in the house, c) money.	Q5: I like how my family and I spend time together.	Always (4 Points)

The range of accumulated scores, for each APGAR test, is between 0 and 20. There is an inversely proportional relationship between the score and the dysfunctionality degree $D_{d}$, i.e., a higher accumulated score generates less perception of family dysfunction. In this way, the interpretation of the score $D_{d}$ is considered as: normal (*N*) if $D_{d}\geq 17$; dysfunctional mild (*M*) if $13\leq D_{g}\leq 16$; dysfunctional moderate (*O*) if $12\leq D_{g}\leq 10$; and severe dysfunctional (*S*) if $D_{g}\leq 9$ [40].

The professional read the questionnaire, considering the score of each answer. The data was collected, cleaned, and unified in a single file with structured format and without missing values. The participants' personal information has been concealed to guarantee their identity remains anonymous. Additionally, those who participated in the project have given their consent for the use of their information for academic purposes, which include scientific journals, presentations, and digital academic repositories.

The dataset has been published in the Harvard Dataverse data repository and is available online, through the following URL: https://doi.org/10.7910/DVN/SW7Q6V.

All the APGAR tests were taken in Spanish since it is the official language of Ecuador, and then transcribed into English. Although the data has been collected by using the APGAR test to measure family functions and their changes in the postmodern context, it is important to note that the collected data could be used for other different purposes.

Since this work aims to explore the changes produced in families, based on the internal perception of their members, an Analysis Of Variance (ANOVA) [47] will be carried out to compare the mean between the degree of perception of family dysfunction at the personal level and collective, in the four categories defined by APGAR questionnaire.

The statistical analysis and data processing was carried out with the programming language, free and open-source software, R v.3.6.1, with the development environment RStudio v.1.2.5040. For the reproduction of the experiments, all the scripts are stored in a public repository, at the link: https://github.com/dievalhu/APGAR.

This research was approved by the ethical committee of the GIFE Research Group of the Universidad Politécnica Salesiana, made up of Ph.D. Robert Bolaños, Ph.D. Jessica Villamar, and Ph.D. Floralba Aguilar.

### 2.4 Procedure

Brief therapy of family accompaniment involves interventions with the family for ten sessions, but in the Ecuadorian context, the family does three to seven sessions, i.e. that the least committed reach three sessions and the most committed ten, so we look for a midpoint that is five sessions. Brief therapy is characterized by having a maximum number of five sessions, with a limited duration of forty-five to sixty minutes per session [48].

The psychology professionals who collected the data received training for five months on the family systemic approach, two months on managing the process, and finally were supervised for another month. The training included the handling of the APGAR test with adults and children. Before applying the APGAR, parents were informed that the questionnaire would be applied to all family members, and children were asked if they wanted to take part. Parents authorized and accompanied the minors during the questionnaire and were assisted by professionals to complete the test if necessary.

The psychology professionals contact the families and explain all the processes in detail. All families provided written informed consent after receiving detailed information. Each family scheduled five home visits. Then, two professionals visit each family; one plays the role of the first-order observer, and the second professional plays the role of second-order observer. A home visit register was done in each visit.

Qualitative methods were applied before carrying out a quantitative method to analyze the results, in order to understand the family system, generate a context of trust, and obtain information that could be more consistent. In this way, with the professionals' accompaniment of the family during several sessions, the family is less likely to hide aspects that would distort the study.

In the first meeting with the family, researchers collected information about the structure of the family using a genogram. This information help professionals to know more about family members and their relationships. The main objective was that the family feel confident with the professionals.

On the second visit, the professionals explore family networks, so they could understand more about the context of the family and how they can be influenced by other systems. In the third meeting, researchers explore family history. The family's past allows knowing the system process.

The fourth home visit had three moments, one to explain the APGAR questionnaire, another to complete the questionnaire, and another for dialogue with the family around the applied questionnaire (a narrative record). The professionals explained the APGAR test to the family, and the members who were at home completed the questionnaire. When they finish the questionnaire, the professional discusses with the family members the issues that APGAR has, so that the family members can talk about their impressions.

Finally, in the fifth meeting, the family remembers all the visits and evaluates how they feel and what they discovered about themselves during the process. They speak about the changes.

There were three home visits to gather information on different aspects of the family system before applying the APGAR test. In this way, the family does not need to simulate the responses, and these can be spontaneous. In addition, family members were in their own context and their responses are observed by psychology professionals. Families are explained that there is no evaluation associated with a medical diagnosis, they also understand that through these home visits they could understand their own family system, get knowledge, and manage changes.

From the qualitative perspective, and after applying the APGAR questionnaire, the professionals spoke with the family members to receive feedback on the statements proposed in the test, the methodological process, and cross-examine to validate the responses posted. In this sense, narratives of some family members were compiled, for instance, when discussing the answers, the women alluded to their role as caregivers of the emotional aspect without this meaning that they received the same affection from other members of the family. Other interesting qualitative elements collected were: the narrative about absences due to work dynamics linked to the use of time; the non-recognition of the proposals of the different members of the family system; and the scarce expression of emotions as a naturalized interaction in the family system.

**Figure 1:**
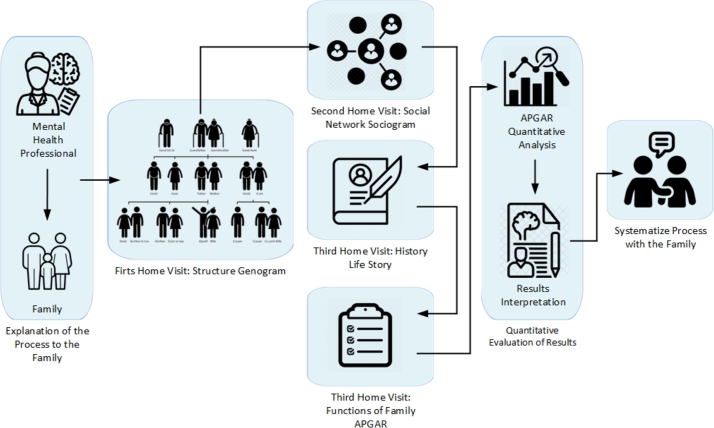
Schematic illustration of the stages implemented in the methodology for the analysis and accompaniment of the family in the postmodern context.

A general outline of the methodology used in this investigation, represented as a block diagram, is shown in Fig. 1.

## 3 Results

This article presents the results of the responses given by family members, collected through the APGAR test, about the functionality of their family system. The results show that the individual and the collective discourse are very similar. On the other hand, the trend is towards a dysfunctional perception of the family system as the years go by and the processes of change concomitant with the postmodern paradigm are accentuated, i.e., the contradictions between the traditional organization existing in the family and the demands from the context.

The first data exploration aims to describe the degree of family dysfunction, evaluated from a collective and individual perspective. For this, the results of the APGAR test have been used as a measurement instrument. In this way, and given the correspondence in the Question-Answer tuple, $Q_{i}-A_{j}$, and their respective weightings, it is possible to numerically evaluate the degree of perception of family and personal dysfunctionality.

Evaluating the APGAR test individually, (n=77), it was found that the general average of the score obtained in the APGAR questionnaire is ${\bar{\mu }}_{D_{d}}=14.48$, which generically determines a mild degree of dysfunction for this sample. Analyzing the taxonomy of dysfunctionality, at the individual level, it is established that 38.96% of the sample is in the normal (*N*) category (class), 36.36% in the mild *M* category, 9.09% in the moderate *O* category and finally 15.59% of the sample in the severe *S* category.

On the other hand, analyzing the data of the family as a unique dynamic system, (n=37), it is observed that the families that have answered the questionnaire have a structure of ${\bar{\mu }}_{m}=2$ individuals on average. The family with the lowest average score in the APGAR questionnaire has a ${\bar{\mu }}_{D_{d}}=4.5$ which places it in the category *S* and the highest average score is ${\bar{\mu }}_{D_{d}}=19.8$, which corresponds to the class *N*, but the average is in class *O* with a ${\bar{\mu }}_{D_{d}}=12.13$. For this evaluation, 43.25% of families were classified in class *N*, 29.73% in class *M*, 13.51% in class *O* and 13.51% in class *S*.

**Figure 2:**
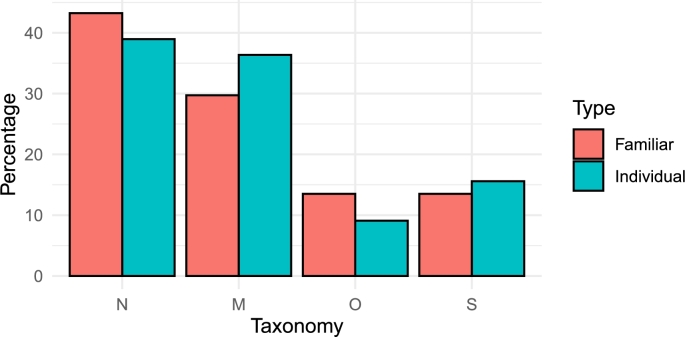
Percentage of dysfunctionality (individual vs. familiar) and its distribution in the four taxonomies.

Fig. 2 shows a percentage representation of the consolidated results, for each of the four dysfunctional taxonomies, based on an evaluation at the individual level and as a family structure.

To know if the perception of dysfunctionality, as an individual, and as a family differ significantly, hypothesis testing was run to contrast the means in the categories, through ANOVA. Thus, we start from the hypothesis that the results of the evaluation of dysfunctionality at the individual and family level do not have differences in any of the established taxonomies (classes), and are represented by the null hypothesis Eq. (1), and the alternative hypothesis Eq. (2).

(1) $$H_{0}:{\bar{\mu }}_{i}-{\bar{\mu }}_{f}=0$$

(2) $$H_{a}:{\bar{\mu }}_{i}-{\bar{\mu }}_{f}\neq 0$$

**Figure 3:**
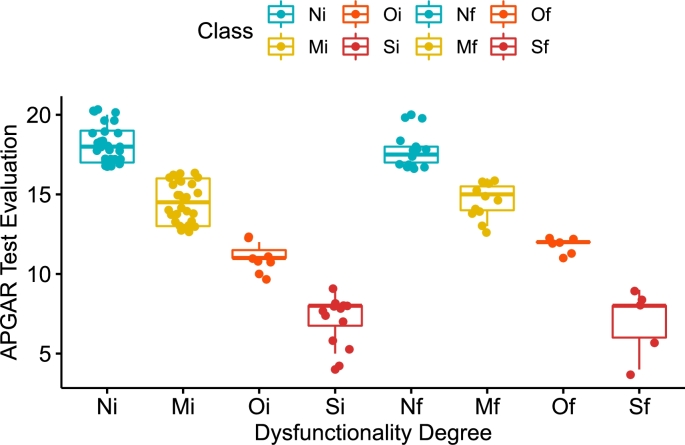
Boxplots of the instances distributed by categories of dysfunctionality: normal (N), mild (M), moderate (O), and severe (S), for the evaluation of Individual (i) and Family (f), in ANOVA testing.

The values to consider in hypothesis testing, for the different taxonomies are: class *N* for individuals (n=30, ${\bar{\mu }}_{N_{i}}=18.13$, $\sigma_{N_{i}}=1.14$) and for families (n=16, ${\bar{\mu }}_{N_{f}}=17.75$, $\sigma_{N_{f}}=1$); class *M* for individuals (n=28, ${\bar{\mu }}_{M_{i}}=14.54$, $\sigma_{M_{i}}=1.23$) and for families (n=11, ${\bar{\mu }}_{M_{f}}=14.64$, $\sigma_{M_{f}}=1.12$); class *O* for individuals (n=7, ${\bar{\mu }}_{O_{i}}=11.14$, $\sigma_{O_{i}}=0.69$) and for families (n=5, ${\bar{\mu }}_{O_{f}}=11.8$, $\sigma_{O_{f}}=0.45$); class *S* for individuals (n=12, ${\bar{\mu }}_{S_{i}}=7.17$, $\sigma_{S_{i}}=1.47$) and for families (n=5, ${\bar{\mu }}_{S_{f}}=7$, $\sigma_{S_{f}}=2$). Fig. 3, shows the box plots of the four classes and their dispersion.

Establishing a significance level of α=0.01, and since the probability values *p*, in the four taxonomies (*N*, *M*, *O* and *S*) are: $p_{N}=0.963$, $p_{M}=0.999$, $p_{O}=0.979$ y $p_{S}=0.999$, respectively; it can be concluded that the $H_{0}$ is accepted and consequently the $H_{a}$ rejected. This implies that there is no significant difference between the perception of dysfunctionality of an individual and a family group.

One of the reasons that could explain why there is no difference between the perception of the dysfunctionality of an individual and a family group, is because, in situations of constant change and uncertainty, members tend to adhere to the discourse of the member with more power or to the dominant story [49]. The pressure by the presence of other family members, while the scale was being applied is also an element that may have influenced the family members to unify their story around dysfunctionality.

It is important to mention that the APGAR test is part of a session in the accompaniment process, in which various topics have been explored with the family. Then, the family member has been learned or reviewed information about the family system and discovers, when applying the test, inconsistencies that lead them to think about dysfunctionality. Thus, as the family talks about its structure, its social networks, and its history, it moves away from the idea of a prototype family, from the western model of the family, and observes the diversity of structures and interactions in the system, which emerge of the traditional model.

The individual and the family seek cohesion from the dominant discourse of dysfunctionality with the desire to return to some certainty, although this return is from the symptom. By persisting in the idea of functionality, from the paradigm of modernity, families, and individuals fall into the diagnosis of dysfunctionality. The individual's actions in daily life are transformed at a different rate than his brain plasticity is transformed, that is, he tries to measure postmodern actions with a modernity paradigm [50].

On the other hand, it is important to analyze the evolution over time of family functions and their changes, since it must be considered that the postmodern context generates changes, and the family also experiences new dynamics, and on many occasions does not know how to process them. Table 2 summarizes the measures of dispersion and central tendency of the data collected, over three years, in the APGAR questionnaires.

**Table 2:** Measures of central tendency and dispersion for the APGAR questionnaires in a temporal line.

Year	Number of questionnaires (*n*)	Evaluation range ($V_{min}-V_{max}$)	Evaluation mean (μ±σ)
2018	23	7 - 20	15.13 ± 4.13
2019	27	6 - 20	14.7 ± 3.47
2020	27	4 - 19	13.7 ± 4.43

The data show a decreasing trend in the average value of the evaluations of the individuals. The perception of individuals regarding the functioning of the family in the current context tends towards difunctionality.

The level of disorder is rising in the family system and the perception of dysfunctionality tends to generalize in the family, as it faces the new challenges of the postmodern context, so it tries to raise entropy to seek a new order in the midst of chaos. In all irreversible processes, the entropy must increase. Therefore, the entropy change in closed systems is always positive, there is continuous destruction of order [51]. The new order is sought from a dysfunctional discourse, i.e., that the family resorts to the symptom, to achieve the confluence of forces that allow the homeostasis of the system [52].

## 4 Discussion

In the 1960s, families underwent multiple changes worldwide, without Ecuador being an exception [53]. Civil society groups questioned the established order at the macro level and proposed legal reforms that did not go unnoticed by the family system. The traditional idea of family is questioned; changes are experienced in an accelerated manner in daily life, moving away from the belief systems that still prevail in families. The internal perception of family functionality denotes the effects of the transition and the risks of resistance to them. Apparently, the postmodern context could be pressuring the family to make changes and it seems that families are not prepared to manage these changes, because they do not have space to observe their own process. One of the changes in the family is related to structure and function.

The objective of this research was to explore if the paradigm of the shift towards post-modernity makes the existing form of organization in the family becomes dysfunctional. In this regard, the first finding of this research shows that the perception of dysfunction at the individual level does not differ from the perception of the family. Likewise, the tendency towards a dysfunctional perception of the family system is evident as the years go by and the processes of change concomitant with the postmodern paradigm become more accentuated. The postmodern family is undergoing several radical changes, involving a redefinition of structure and functions to adapt to the context and its needs [54].

The stress caused by changes in the family can lead to confusion or incoherence in the social context of families. In relation to this last idea, it is important to consider that institutions, such as school, work, church, health services, also experience a crisis because they encounter a family that is not aligned with their concepts. Institutions also continue to use definitions of family that belong to modernity. For example, the family network could put pressure on the family to return to the old paradigm because the change in family systems also means a change in institutional contexts. Institutions are forced to perform functions that belong to family systems, so perhaps this is one of the reasons why family systems are considered dysfunctional [55].

Since there are no similar studies in Ecuador, we have considered studies that have some relationship with family studies. In a study conducted in the United Arab Emirates [62], which is one of the cultures currently adopting the customs of Western culture, generational and cultural changes in family life were investigated. The results coincide with those of the research, showing that the postmodern context increases dysfunctions in the family system.

So also, in a research conducted by [56] that aimed to know the family time with the parents of 17 dual-income children and 11 single-parent families, the results showed that, although the families have a positive expectation of being together, they feel that the family is at the service of the children. There is a structural contradiction between ideals and the experience of family time that is typically expressed through disappointment and guilt. These results relate to those obtained in this research, where; the long-term postmodern context could increase dysfunctions in the family system. Finally, Daly's study shows that families monitor conflict and what family role change entails, but do not talk about it because they do not know how to handle the change.

The concept of family has changed, and it is evident that cultural differences have emerged, but apparently in everyday life, the way of understanding the family is still aligned with the paradigm of the modern family, so families begin to feel dysfunctional because they have not assimilated the new concepts of family [57]. They live the changes, but they do not make a process to manage the changes.

It is important to understand that the family naturally tries to avoid change, because within the family change is associated with crisis, pain, risks that the family cannot manage, because they are risks in fields in which the family has not developed skills [58].

On the emotional level, it is essential to consider that labor dynamics and new forms of work have implied the current absences of parents in the family context with the result of dissatisfaction related to psychological needs [55]. Perhaps this could explain why violence is a constant in family systems. The emotional process is repressed in family systems, parents feel guilty and children feel abandoned, which represents a circuit that reinforces the idea of dysfunction [59].

In this sense, according to research by [60], children who live with their married biological parents consistently have higher physical, emotional and academic well-being compared to children who live with parents who are not married or who live with a single member of the paternal or maternal figure. These results are somewhat related to those obtained in this research, since it is confirmed that the postmodern context promotes a change in the functions of the family, but that the family does not talk about it, although they know that the modern era and its implications gave them a certain stability.

The family is a complex institutional structure that interweaves experiences, emotions, and interactions among its members. Postmodernism has challenged the system and, therefore, families to adapt to new situations and profound changes, where the perception of dysfunctionality in some families is the new normal. This new understanding merits the strengthening of psycho-social, emotional, and communication skills among all family members.

### 4.1 Limitations and future directions

The findings that were found in our research are important from a systemic perspective that helps to understand the functions of the family. But some of the limitations should be mentioned. The information presented in this study corresponds to the fourth follow-up home visit to families and shows only quantitative and not qualitative data. Furthermore, resource constraints have not allowed extended to other places or compared with other cultures, this research. The study is cross-sectional and therefore the direction of causality cannot be deterred. Although the study was carried out in different years, it was not carried out on the same families, therefore, the coincidence between the perception of dysfunctional at the individual and family level could be due to specific factors such as the psycho-emotional health of its members and the experiences lived and inherited from the same family. In addition, this study of changes in family functions does not consider an evaluation per se of the families and their perception in the interaction with the different institutions, such as the state, the school, work, marriage, etc.

It should also be considered that the study was carried out with a sample of families collected in a relatively short period of time and with only the APGAR questionnaire as a measurement instrument. Therefore, it is not possible to affirm that there are changes in family functions in the transition to postmodernity within a historical process, but rather the quantitative results of this research show that this trend is increasing.

Future research may include interviews with families about their interaction with the institutions to which they belong to determine the level of influence in the understanding of family functions, in addition to incorporating measures of stress to the family members, since can be considered as a risk factor or a protection factor [61]. Knowing this aspect would determine the understanding to efficiently manage the changes and conflicts that occur in family dynamics.

Some prospects for this work also include processing the information collected in the first visits to the family to contrast with the data presented in this first quantitative analysis; process data around each family function and contrast with qualitative data obtained in the same session; and collect information in other sectors and compare them with ethnic-cultural variables.

## 5 Conclusion

This article examines family functions in a postmodern society, analyzed quantitatively using the APGAR questionnaire, during three years in the Ecuadorian context. The quantitative results have shown that the differences in dysfunctional perception between the individual and the family system are statistically similar. Besides, it is shown that the temporal evolution to postmodernity, numerically evidences that the perception of family dysfunctional is exacerbated. The results suggest that it is necessary to create mechanisms to help families to adapt to the new paradigm.

In future works, we would like to analyze the results of the relation between the APGAR test and other variables that are associated with changes in the postmodern context.

## Declarations

### Author contribution statement

Catya Torres, Diego Vallejo-Huanga: Conceived and designed the experiments; Performed the experiments; Analyzed and interpreted the data; Contributed reagents, materials, analysis tools or data; Wrote the paper. Ximena Ramırez Ocaña: Conceived and designed the experiments; Contributed reagents, materials, analysis tools or data; Wrote the paper.

### Funding statement

This work was supported by the 10.13039/100016969Salesian Polytechnic University, Ecuador (Phase III Machine Learning Applications, 2020).

### Data availability statement

Data associated with this study has been deposited at the Harvard Dataverse Repository: https://doi.org/10.7910/DVN/SW7Q6V.

### Declaration of interests statement

The authors declare no conflict of interest.

### Additional information

No additional information is available for this paper.
